# Driver Distraction Using Visual-Based Sensors and Algorithms

**DOI:** 10.3390/s16111805

**Published:** 2016-10-28

**Authors:** Alberto Fernández, Rubén Usamentiaga, Juan Luis Carús, Rubén Casado

**Affiliations:** 1 Grupo TSK, Technological Scientific Park of Gijón, 33203 Gijón, Asturias, Spain; juanluis.carus@grupotsk.com; 2 Department of Computer Science and Engineering, University of Oviedo, Campus de Viesques, 33204 Gijón, Asturias, Spain; rusamentiaga@uniovi.es (R.U.); rcasado@lsi.uniovi.es (R.C.)

**Keywords:** driver distraction detection, visual-based sensors, image processing

## Abstract

Driver distraction, defined as the diversion of attention away from activities critical for safe driving toward a competing activity, is increasingly recognized as a significant source of injuries and fatalities on the roadway. Additionally, the trend towards increasing the use of in-vehicle information systems is critical because they induce visual, biomechanical and cognitive distraction and may affect driving performance in qualitatively different ways. Non-intrusive methods are strongly preferred for monitoring distraction, and vision-based systems have appeared to be attractive for both drivers and researchers. Biomechanical, visual and cognitive distractions are the most commonly detected types in video-based algorithms. Many distraction detection systems only use a single visual cue and therefore, they may be easily disturbed when occlusion or illumination changes appear. Moreover, the combination of these visual cues is a key and challenging aspect in the development of robust distraction detection systems. These visual cues can be extracted mainly by using face monitoring systems but they should be completed with more visual cues (e.g., hands or body information) or even, distraction detection from specific actions (e.g., phone usage). Additionally, these algorithms should be included in an embedded device or system inside a car. This is not a trivial task and several requirements must be taken into account: reliability, real-time performance, low cost, small size, low power consumption, flexibility and short time-to-market. The key points for the development and implementation of sensors to carry out the detection of distraction will also be reviewed. This paper shows a review of the role of computer vision technology applied to the development of monitoring systems to detect distraction. Some key points considered as both future work and challenges ahead yet to be solved will also be addressed.

## 1. Introduction

According to the most recent published World Health Organization (WHO) report, it was estimated that, in 2013, 1.25 million people were killed on the roads worldwide, making road traffic injuries a leading cause of death globally [1]. Most of these deaths happened in low- and middle-income countries, where rapid economic growth has been accompanied by an increased motorization and therefore, road traffic injuries. In addition to deaths on the roads, up to 50 million people incur non-fatal injuries each year as a result of road traffic crashes, while there are additional indirect health consequences associated with this growing epidemic. Road traffic injuries are currently estimated to be the ninth leading cause of death across all age groups globally, and are predicted to become the seventh leading cause of death by 2030 [1].

Distracted driving is a serious and growing threat to road safety [1]. Collisions caused by distracted driving have captured the attention of the US Government and professional medical organizations during the last years [2]. The prevalence and identification as a contributing factor in crashes is seen as an *epidemic* of American roadways, in words of Ray LaHood, when he was US Secretary of Transportation in 2012 [3]. There is not an exact figure regarding statistics about accidents caused by inattention (and its subtypes) since studies are made in different places, different time frames and therefore, different conditions. The studies referenced below show both the different statistics about inattention in general and those recorded when produced by distraction and fatigue in particular. These authors have estimated that distraction and inattention account for somewhere between 25% and 75% of all crashes and near crashes [4,5,6,7,8].

The trend towards increasing the use of in-vehicle information systems (IVISs) is critical [9] because they induce visual, manual and cognitive distraction [10] and may affect driving performance in qualitatively different ways [11]. Additionally, the advancement and prevalence of personal communication devices has exacerbated the problem during these last years [12]. All these factors can lead to the increment of the number of tasks subordinate to driving activity. These tasks, namely secondary tasks, which may lead to distraction [13], include eating, drinking, the act of taking something or tuning the radio or the use of cell phones and other technologies. The secondary tasks that take drivers’ eyes off the forward roadway [14,15] reduce visual scan [16] and increase cognitive load may be particularly dangerous [13]. For example, the use of cell phones while driving, according to naturalistic studies [17], causes thousands of fatalities in the United States every year [18,19].

**Figure 1:**
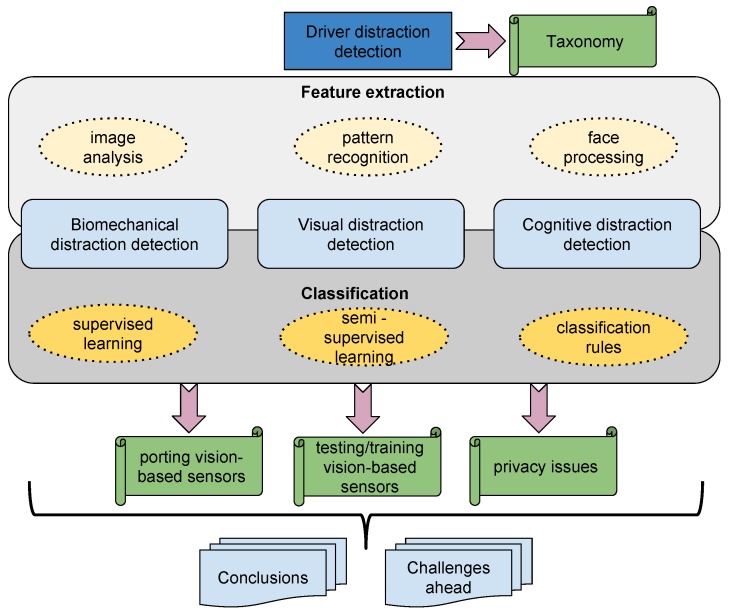
Scope of the present work.

The purpose of this paper is the analysis of the state-of-the-art regarding the detection of drivers’ distraction. The scope of the paper can be seen in Figure 1 and is commented as follows. The main methods for face detection, face tracking and detection of facial landmarks are summarized in Section 2 because they are a key component in many of the video-based inattention monitoring systems. In Section 3, Section 4 and Section 5, the main algorithms for biomechanical, visual and cognitive distraction detection are reviewed, respectively. Additionally, in Section 6, there are some algorithms detecting mixed types of distraction and, hence, are also reviewed. The relationship between facial expressions and distraction is also explored in Section 7. The key points for the development and implementation of sensors to carry out the detection of distraction will be considered in Section 8. In Section 9, the key ones to test and train driving monitoring systems are summarized. Privacy issues related to camera sensors are commented in Section 10. Lastly, conclusions, future aspects and challenges ahead will be considered in Section 11.

With the objective of introducing the scope and limitations of this review, some key aspects have been briefly introduced as follows. Driver distraction is just one form of inattention, which occurs when drivers divert their attention away from the driving task to focus on another activity. Therefore, a “complete” solution should consider all aspects of inattention. At least, the system should detect both distraction and drowsiness as the main contributing factors in crashes and near-crashes. As stated before, in this work, only distraction algorithms are summarized but one must not forget that other forms of inattention should be taken into account. Moreover, the use of on-board sensors already available in the vehicle to analyze driver behaviour is a low-cost and powerful alternative to the vision-based monitoring systems [20,21]. However, these systems should not be treated like different alternatives, because they can be used together (fusioned) in order to obtain indicators for monitoring [22]. Hence, for the sake of completeness, in this paper review only “purely” vision-based monitoring systems have been reviewed.

One of the challenges in decreasing the prevalence of distracted drivers is that many of them report that they believe they can drive safely while distracted [23]. However, for example, in connection with the use of mobile phones while driving, there is a great deal of evidence interacting with mobile devices, such as sending messages or engaging in conversations, which can impair driving performance because this interaction can create distraction. Moreover, a recent research showed that phone notifications alone significantly disrupted performance, even when drivers did not directly interact with a mobile device during the task [24]. Another study suggests that people in general can reduce both inattention and hyperactivity symptoms simply by silencing the smartphones and avoiding notifications [25]. Therefore, it is clear that drivers should not use and notice the presence of the smartphones inside the car while driving. It should be pointed out that distraction generation is a very complex process and is scarcely addressed here. We recommend some research papers that focused on driver distraction generation: Angell et al. [26] focused on the process of cognitive load in naturalistic driving; Liang et al. [27] addressed the adaptive behaviour of the driver under task engagement and their results on visual, cognitive and combined distraction; Caird analyzed the effects of texting on driving [28]. In the context of intelligent vehicles, Ohn et al. [29] highlights the role of humans by means of computer vision techniques.

### 1.1. Taxonomy

Both distraction and inattention have been inconsistently defined and the relationship between them remains unclear [30]. The use of different, and sometimes inconsistent, definitions of driver distraction can create a number of problems for researchers and road safety professionals [31]. Inconsistent definitions across studies can make the comparison of research findings difficult or impossible, can also lead to different interpretations of crash data and, therefore, to conclude different estimates of the role of distraction in crashes. This problem can be further seen in these recent works [32,33,34,35]. Many definitions have been proposed in order to define distraction [5,7,8,31]. Regan et al. [35] proposed a taxonomy of both driver distraction and inattention in which distraction is conceptualized as just one of several factors that may give rise to inattention. They concluded that driver inattention means “*insufficient or no attention to activities critical for safe driving*”. They defined driver distraction as “*the diversion of attention away from activities critical for safe driving toward a competing activity, which may result in insufficient or no attention to activities critical for safe driving*”. The definition proposed here is almost identical to that coined for driver distraction by Lee et al. [31].

It is acknowledged that the taxonomy proposed by Reagan et al. [35] suffers from “hindsight bias”, that is, the forms of driver inattention proposed are derived from studies of crashes and critical incidents in which judgements have been made after the fact about whether or not a driver was attentive to an activity critical for safe driving [35]. Driving consists of a variety of sub-tasks and it may not be possible to attend to all at the same time. Determining which sub-task is more important (and the driver, thus, should attend to) can often only be determined after the fact (i.e., after a crash or incident occurred) and, hence, this attribution of inattention is somewhat arbitrary [36]. Additionally, the dynamics of distraction [37], which identifies breakdowns on interruption as an important contributor to distraction should also be considered as part of this taxonomy, and hence, timing and context have implications on the algorithm design that should be taken into account.

### 1.2. Methodology

Papers addressed in this review are within the topic of distraction detection using vision-based systems. The search and review strategy is described below. A comprehensive review of the English language scientific literature was performed. It encompassed the period from 1 January 1980 to 31 August 2016. The following databases were used: EBSCO, ResearchGate, ScienceDirect, Scopus, Pubmed, Google Scholar and Web of Knowledge. Search terms related to driver distraction were employed combining all of them: driver, visual, cognitive, manual, biomechanicall, vision, vision-based, impairment, distraction, distractions, review, task, tasks, inattention, performance, phone, sms, vehicle, problem, looking, face, head, pose, glasses, illumination, self-driving, tracking, sensors, image, traffic, safety, facts, privacy, issues, porting, taxonomy. Many items were returned from the search criteria shown before. These were, then, reviewed using the following criteria. Exclusion criteria were obviously non-relevant papers or from medical, electronic, networking, marketing and patent topics. Only publications from peer-reviewed English language journals were considered for inclusion. Additionally, reviewed papers were ordered by the number of references in order to include all relevant papers. Finally, in order to get the latest published papers, search filters were applied for this purpose. Search filters were applied to get publications only from years 2015 and 2016. References and bibliographies from the selected papers identified were examined to determine potentially additional papers. A total of approximately 1500 publications were revised in the review process.

## 2. Face and Facial Landmarks Detection

**Figure 2:**
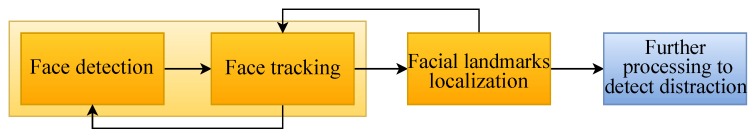
Common steps in most distraction monitoring systems.

A common face processing scheme in many inattention monitoring systems, which can be seen in Figure 2, includes the following steps:

- Face detection and head tracking. In many cases a face detection algorithm is used as a face tracking one. In other cases, a face detection algorithm is used as an input for a more robust face tracking algorithm. When the tracking is lost, a face detection call is usually involved (that is why in Figure 2 these steps are placed inside the same external orange box).
- Localization of facial features (e.g., eyes). Facial landmarks localization is usually performed, but it should be noted that, in some cases, no specific landmarks are localized. So, in such cases, estimation of specific cues are extracted based on anthropometric measures from both face and head.

### 2.1. Face Detection

Viola-Jones [38] have made object detection practically feasible in real world applications, which contains three main ideas that make possible to build and run in real time: the integral image, classifier learning with AdaBoost, and the attentional cascade structure [39]. This framework is used to create state-of-the-art detectors (e.g., face detector [40]), available, for example, in Opencv library. However, this framework turned out to be really time-consuming [41]. Moreover, cascade detectors work well on frontal faces but sometimes, they fail to detect profile or partially occluded faces.

One possible solution is to use the standard approach for human detection [42], which can also be used for face detection [39]. This approach is based on the Histogram of Oriented Gradients (HOG), which is a feature descriptor used in computer vision and image processing for the purpose of object detection. This approach can be trained with less images and faster [43]. Deep Learning approaches can also be used for face detection. For example, in [44], a deep learning approach, called DP2MFD, is used. DP2MFD detects faces at multiples scales, poses and occlusion by integrating deep pyramid features with Deformable Parts Models (DPMs). Experiments were carried out on four publicly available unconstrained face detection datasets, which demonstrated the effectiveness of the approach. However, this face detector was tested on a machine with 4 cores, 12 GB RAM, 1.6 GHz processing speed and it took about 26 s. Consequently, complex features may provide better discrimination power than Haar-like features for the face detection task. However, they generally increase the computational cost [44].

Some modifications to the Viola-Jones algorithm have been proposed [45,46] to speed up the algorithm. For example, in [45], different optimization techniques to speed up the Viola-Jones detector for embedded smart camera applications have been discussed. In their paper, skin colour information is integrated with the Viola-Jones detector in order to reduce the computation time. PICO (Pixel Intensity Comparison-based Object detection) is another modification of the standard Viola-Jones object detection framework, which scans the image with a cascade of binary classifiers at all reasonable positions and scales [46]. This algorithm can achieve competitive results at high processing speed. This is especially evident on devices with limited hardware support for floating point operations. PICO outperforms the other two OpenCV detectors in terms of accuracy and processing speed.

Since driver face monitoring system should work in all light conditions, lighting and camera selection is one of the most important stage in the design of the system. Lighting devices not only should provide enough light in environment, but they also should not hurt his/her eyes. For example, learning-based methods (e.g., Viola-Jones algorithm or PICO) can also be used for face detection in Infrared (IR) images [47].

Position of the camera inside the car is another key factor in the detection rate. For example, in [48], if the camera is installed under the front mirror of the car, face detection has 85% accuracy. But if it is installed on the dashboard, face detection reaches up to 93%. This is because they used the Viola-Jones face detector, which is trained to distinguish faces that are tilted up to about 45${}^{\circ }$ out of plane (towards a profile view) and up to about 15${}^{\circ }$ in plane. Therefore, if the camera is installed on the dashboard, the captured images will contain frontal or near-frontal faces. In [49], the camera was placed over the steering wheel column for two reasons: a) it facilitates the estimation of gaze angles, such as pitch, which is relevant for detecting distraction, and b) from a production point of view, it is convenient to integrate a camera into the dashboard. On the downside, when the wheel is turning, there will be some frames in which the drivers face may be occluded by the steering wheel. However, the driver is seldom very sleepy or inattentive to traffic while turning the steering wheel.

### 2.2. Face Tracking

**Figure 3:**
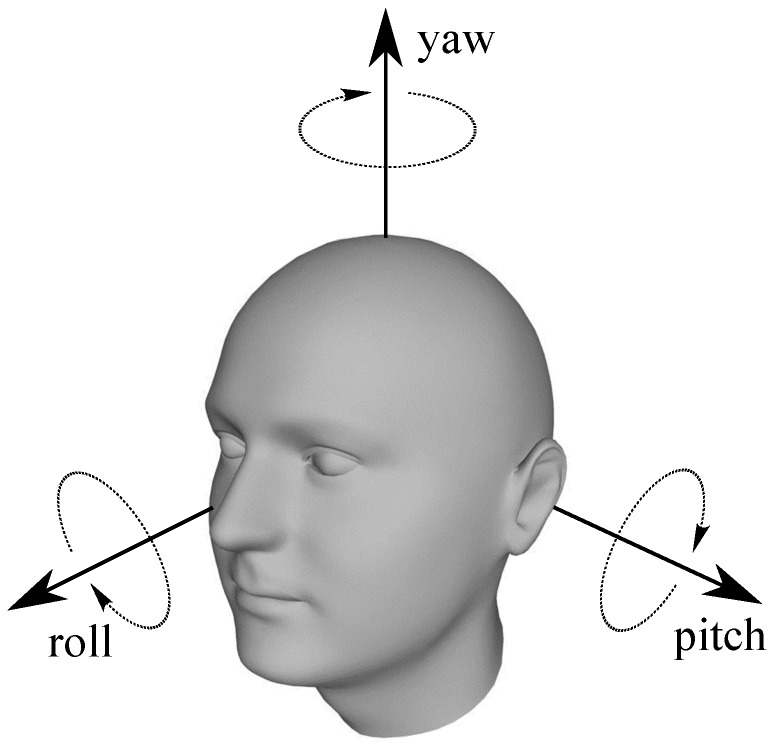
Head pose can be decomposed in pitch, yaw and roll angles.

Head pose estimation can be defined as the ability to infer the orientation of a person’s head relative to the view of a camera and different studies have reported statistics showing consistent range of head motion [50], which (see Figure 3) can be decomposed in:

- Saggital flexion/extension, i.e., forward to backward movement of the neck usually from −60° to 70°, which can be characterized by pitch angle.
- Axial rotation, i.e., right to left rotation of the head usually from −80° to 75°, which can be characterized by yaw angle.
- Lateral bending, i.e., right to left bending of the neck usually from −41° to 36°, which can be characterized by roll angle.

Many vision-based algorithms for pose estimation have shown good performance when the head is near frontal, which is 95% of the time. But it is during those 5% of the time when interesting events, which are critical for safety, will occur [51]. Furthermore, as face orientation while driving is normally frontal, if the driver faces in other directions for a long period of time, this is probably due to fatigue or inattention [52]. Hence, a key component for a monitoring system based on face tracking is the ability to robustly and continuously operate even during large head movements. However, face tracking remains a challenging vision problem and, hence, a system for a continuous estimation of head movement is needed. On the other hand, as many head tracking algorithms have shown good performance when the head is near frontal, it can be concluded that the driver is looking away when tracking is unavailable. This information could be an alternative approach instead of adding more cameras to increase the range of the tracker.

Accordingly, numerous research works and publications have been trying to perform face tracking using a single camera and they are discussed as follows. Head pose estimation methods based on geometric approach using facial landmark and its 3D correspondences [49,53,54,55,56] can provide a good estimation and operate in real-time. For example, in [53], 3D pose estimation is achieved based on the position of the eyes and the mouth. A similar approach is proposed in [54], where only three points (eye centers and the middle point between the nostrils) are used to estimate continuous head orientation and gaze direction. Very closed to this approach, in [55], at least four prominent facial features are extracted from the face. After that, their correspondence on a 3D generic-face model is used to estimate head orientation. Oyini et al. [56] proposed the visual analysis of head position using a single camera aligning and scaling the 3D head model of the face according to the position and distance between the two eyes of the face in the 2D image. Another interesting approach recently published is [49], where a 3D head pose estimation system is proposed. This system is based on the 49 tracked 2D facial landmarks from Supervised Descent Method (SDM) tracker [57].

Other options include the combination of information [58,59,60], using for example, several classifiers [58,59] or combining 2D and 3D algorithms [60]. Asthana et al. [58] developed a system able to handle 3D pose variations up to ±45° in yaw and ±30° in pitch angles combining four different face detectors based on Viola-Jones framework. The drawback of this approach is that it requires four classifiers in order to track the face so it increases the execution time and memory requirements. In [59], the system consists of three interconnected modules, which detects drivers’ head, provides initial estimates of head pose, and continuously tracks its position and orientation in six degrees of freedom. Pelaez et al. [60], combined 2D and 3D algorithms to provide head pose estimation and regions of interest identification based on 3D information from a range imaging camera.

Alternatively, more than a camera can be used to implement the tracking [51,61,62,63], that is, a distributed camera system is commonly used, where two or more cameras can be located inside the car cockpit. Following this line of research, in [61], they proposed a distributed camera framework for gaze estimation using head pose dynamics based on the algorithm proposed in [51]. They predict three gaze zones: right, front and left. In [51], a continuous head movement estimator (CoHMEt) is proposed, which independently tracks the head in each camera, and their outputs are further analyzed to choose the best perspective and corresponding head pose. When tracking is lost, due to either the loss of facial point detection or the rejection of the estimated points, reinitialization is performed using a scoring criterion. In [62], they also used a two-camera system to overcome challenges in head pose estimation, which allows for continuous tracking even under large head movements, as proposed in [51]. Therefore, following the setup of [51] , a two-camera system can provide a simple solution in order to improve tracking during large head movements. Two cameras are also used in [63] for head pose estimation. Head pose is tracked over a wide operational range in the yaw rotation angle using both camera perspectives.

For a quantitative evaluation over the algorithms the Mean Absolute Error (MAE) is commonly used. Best results for the different algorithms can be seen in Table 1, where different databases are used. For example, in [49,56] the public database of Boston University (BU) is used to evaluate the performance of the proposed head pose estimation scheme. Some other algorithms used naturalistic on-road data set [59]. Moreover, some algorithms achieving good performance did not indicate any database [60]. LISA-P Head Pose database [55] introduces head pose data from on-road daytime and nighttime drivers of different age, race and gender, with continuous ground truth measurements and manual annotation of facial features. Therefore, this database can be used to compare head pose algorithms and head behaviour studies. The LISA-P Head Pose Database consists of 14 video sequences of drivers in on-road driving environment in natural and spontaneous conditions. The video sequences were collected at a frame rate of 30 frames per second, with a 640 × 480 pixel resolution.

Based on the results from Table 1, in [56], the MAE decreased by an average of 1.3° due to illumination variations. In [51], the best performance of 3.9% failure rate, which is the percentage of the time that the system output is unreliable, is achieved with the three-camera view compared with that of over 15% for the single view, which is a significant improvement.

### 2.3. Location of Facial Features

The detection of facial features (also called landmarks) is an essential part of many face monitoring systems. The problem of the precise and robust detection of facial landmarks has drawn a lot of attention during this decade. State-of-the-art methods include tree models [65,66], DPM [67], SDM [57], explicit shape regression [68] or learning local binary features [69]. A comprehensive survey of facial feature point detection can be seen here [70]. All the above listed research suffers more or less from a lack of verification and performance analysis with a realistic variation in lighting conditions. Therefore, further research should be performed in order to adapt these algorithms to the traffic research in general and to the drivers’ monitoring systems in particular. Difficulties for proper detection of drivers’ facial features are mainly due to the non-uniformity of light sources, asymmetric shades on their face and eye regions, or rapid changes in light intensity during real-world driving due to shadows caused by buildings, bridges, trees, or, for example, when entering or leaving a tunnel [71].

Eyes, as one of the most salient facial features reflecting individuals’ affective states and focus of attention [72], have become one of the most remarkable information sources in face analysis. Eye tracking serves as the first step in order to get glance behaviour, which is of most interest because it is a good indicator of the direction of the driver’s attention [73]. Glance behaviour can be used to detect both visual and cognitive distraction [74]. It has also been used by many studies as an indicator of distraction while driving [75] and has been evaluated in numerous ways [73]. Therefore, both eye detection and tracking form the basis for further analysis to get glance behaviour, which can be used for both cognitive and visual distraction.

Eye tracking data is typically captured through the use of a vehicle instrumented with an in-vehicle eye tracker system. On one hand, complex systems consist of single or multiple cameras directed at the driver’s face. As the number of face cameras increases, so does the ability of the system to capture larger and more dramatic head movements of the driver. On the other hand, simpler systems consisting of one or two cameras are usually less expensive and easier to install than more complex systems. For example, in [76], a comparison of eye tracking systems with one and three cameras using Smart Eye technology [77] is performed. The system uses a single standard camera of VGA resolution together with IR flash illuminators. The three-camera system used is the Smart Eye Pro [77], which has similar properties as the one-camera system, but it also facilitates gaze direction in full 3D.

Eye detection is required before eye region processing. Eye detection methods can be divided into two general categories: (1) methods based on imaging in IR spectrum; and (2) feature-based methods. A literature survey on robust and efficient eye localization in real-life scenarios can be seen in [72], and a review on eye localization in car environment can be seen in [78].

Methods based on imaging in IR spectrum, which are commonly called “hardware-based” approaches, rely on IR illuminators to generate the bright pupil effect to driver head pose and gaze estimation. These methods use two ring-type IR light-emitting diodes: one located near the camera optical axis and the other located far from it. This approach is often used to detect visual distraction. In contrast to these methods, in [79], the authors use a progressive camera and only one on-axis lighting source [80]. In this situation, the camera always produces images with bright pupils and image processing techniques are applied to detect pupils. Based on thresholding techniques, the possible pupils can be selected. An appearance model, trained using Principal Component Analysis (PCA) and Support Vector Machine (SVM), is exploited to verify the final pupils. To increase the robustness against eyeglasses, the Generalized Symmetry Transform (GST) is incorporated achieving a recognition rate of 99.4% and 88.3% for users not wearing and wearing eyeglasses, respectively.

Regarding feature-based methods, different techniques are commonly applied. Image binarization [81], projection [82,83], face anthropometric properties of the face [84], individual classifiers [85] or particle filtering [86] can be used to detect driver’s eyes. For example, in [86], an algorithm for eyes tracking based on particle filtering is proposed. Their method works with a low-cost IR camera device at a low frame rate. They used a single particle filter to track both eyes at the same time. Evaluation was carried out in a driving simulator with five users achieving an average accuracy of 93.25%. In [85], two individual classifiers based on Haar-like features, one for the head and another for both eyes, were used. They tested face and eye detection in their research vehicle in daylight conditions achieving a hit rate of 97.2% for eye detection and a false alarmn of 4.6%.

All in all, the task of accurate eye localization is challenging due to the high degree of eyes appearance variability: facial expression variations, occlusion, pose, lighting and other imaging conditions and quality [72], are frequently encountered in car environments. Another problem that is scarcely addressed in the literature is that, in strong sunlight, the driver tends to squint, which makes, even more difficult to track the eyes. To mitigate these deficiencies, different approaches can be adopted. Sigari et al. [82] proposed to extract symptoms of hypo-vigilance based on eye-region processing but without explicit eye detection stage. Flores et al. [84] proposed a combination of algorithms in order to deal with illumination conditions for both day and night. Rezaei et al. [71] used a methodology to enhance the accuracy, performance and effectiveness of Haar-like classifiers, especially for complicated lighting conditions. These authors also proposed ASSAM [87], which is based on the asymmetric properties of the driver’s face due to illumination variations. A good solution is also to use a “divide and conquer” strategy to handle different variations at different stages [72].

## 3. Biomechanical Distraction

In connection with biomechanical detection and recognition using computer vision techniques, we can find two approaches. The first one involves hands secondary tasks recognition involving hands action, while the second one is based on hands tracking and information.

### 3.1. Secondary Tasks Involving Biomechanical Distraction

Zhao et al. [88,89,90,91] proposed different maching learning approaches to detect predefined driving postures, where four predefined postures were considered: (1) grasping the steering wheel; (2) operating the shift lever; (3) eating; and (4) talking on a cellular phone, which are recorded from the passenger seat, that is, from the right profile view of the driver. Yan et al. [92] proposed a combination of the Motion History Image (MHI) and POHG, and the application of Random Forest (RF) classifier for driving actions recognition. Trying to improve the accuracy of the aforementioned approach, the same authors included a Convolutional Neural Network (CNN) [93], which was tested over three datasets covering four driving postures: (1) normal driving; (2) responding to a cell phone call; (3) eating; and (4) smoking. For fair comparison, Yan et al. [93] re-implemented aforementioned state-of-the-art approaches [88,89,90,91] and carried out experiments on other two popular vision descriptor approaches (PHOG [94] and SIFT [95]). Classification accuracy of all of these methods can be seen in Table 2 evaluated on the Southeast University (SEU) driving posture dataset [88].

In connection with secondary tasks recognition, different computer vision algorithms have been proposed in order to detect cell phone usage of the driver while driving [96,97,98,99,100]. High recognition rates are usually obtained (from 86.19% to 95%) using very different approaches. Computer vision techniques seem to be the best approach for this task, whose results can be seen in Table 3, compared to other non-computer vision algorithms relying on inertial sensors of the mobile phone [101]. Best results are obtained by the algorithm proposed by Xu et al. [99], which consists of two stages: first, the frontal windshield region localization using DPM; next, they utilized Fisher vectors (FV) representation to classify the driver’s side of the windshield into cell phone usage violation and non-violation classes. The proposed method achieved about 95% accuracy with a dataset of more than 100 images with drivers in a variety of challenging poses with or without cell phones.

It can be concluded that many different computer vision and machine learning techniques can be used to recognize predefined postures involving hand gestures. The CNN model offered a better performance than other approaches but with some limitations. The algorithm needs high computational resources making difficult to be applied in some conditions with common hardware architecture (e.g., embedded systems). Moreover, training a CNN needs a large amount of data, which is also difficult to obtain in some scenarios.

### 3.2. Hands Information

Hand detection is a challenging problem as human hands are highly deformable and are also exposed to different illumination conditions [102]. One approach for object detection relies on a sliding-window, where a model is learned based on positive samples (i.e., hands in different poses) of fixed size and negative samples with no hands. A classifier is then used to learn a classification rule. In order to detect hands at different scales, this scheme can be applied on hand images at different sizes. But a sliding window-based approach trained on hand instances was shown to be prone to false positive detection rates [103]. A recent common approach to improve the results is the assumption that hands can only be found in a small and predefined set of regions [103,104].

As opposed to training a model for hand shape or appearance and running a sliding window detector, two different approaches are analyzed in [103] taking into account three activity classes: (1) two hands on the wheel; (2) hands on the instrument panel and (3) hand on the gear shift. The motion-cue-based hand approach uses temporal accumulated edges in order to maintain the most reliable and relevant information motion and then, it is fitted with ellipses in order to produce the location of the hands. The static-cue-based approach uses features in each frame in order to learn a hand presence model for each of the three regions and a second-stage classifier (SVM) produces the final activity classification. Martin et al. [104] also constraint the problem of hands detection to a number of regions of interest. They used HOG at different scales. Afterwards, a SVM is used to learn a hand presence in each of the three regions and ‘two hands on the wheel’ model for the wheel region. A similar approach is proposed in [102], training a linear SVM model for each region using a different set of descriptors.Ohn et al. [62] incorporated hand gestures in order to study preparatory motions before a maneuver had been performed, training a hand detector using fast feature pyramids. Gradient and colour channels are extracted for each patch image. They used CIE-LUV colour channels because they worked better compared to RGB and HSV. Afterwards, an AdaBoost classifier was applied in order to learn the features from the hands and finally, they trained a SVM-based detector using HOG features to differentiate the left hand from the right one. Later on, Ohn et al. [105] also explored the use of a pyramidal representation for each region of interest using HOG finding that edge features are particularly successful in the task of hands detection.

In order to compare these algorithms, a dataset of synchronized RGB and depth videos collected in an operating vehicle was proposed [106]. The CVRR-HANDS 3D dataset was designed in order to study natural human activity under difficult settings (background, illumination, occlusion) containing three subsets: (1) hand localization; (2) hand and objects localization; and (3) 19 hand gestures for occupant-vehicle interaction. Five regions of interest were considered: (1) wheel; (2) lap; (3) hand rest; (4) gear; and (5) instrument panel. Recognition rates from some of these previous algorithms using this database can be seen in Table 4.

Summarizing, a common approach is to recognize if the hands are positioned in one of the established areas (wheel, gearbox and so on) and to track them over time. It could be considered that the steering wheel is the critical area because it is where hands should remain most of the time while driving. If hands remained in a non-critical zone for a certain period of time, which could be different for each of the non-critical areas, an alarm would be created to warn drivers to lay their hands in the correct position.

#### Hand Disambiguation

There is another interesting problem to solve related to hands detection that needs further research: hand disambiguation [107]. Once hands are detected, it is crucial to ensure that the hands belong to the driver. Both hand disambiguation and hand activity detection should be studied and considered together in order to infer final, clear and unambiguous results.

## 4. Visual Distraction

Visual distraction is often related to the on-board presence of electronic devices such as mobile phones, navigation or multimedia systems, requiring active control from the driver. It can also be related to the presence of salient visual information away from the road causing spontaneous off-road eye glances and momentary rotation of the head. A 2006 report on the results of a 100-car field experiment [4] showed that almost 80% of all crashes and 65% of all near-crashes involved drivers looking away from the forward roadway just prior to the incident.

Engagements in visually distracting activities divert drivers’ attention from the road and cause occasional lapses, such as imprecise control of the vehicle [108], missed events [28], and increasing reaction times [108]. Visual time sharing between the driving task and a secondary task reveals that the glance frequency to in-car devices is correlated to the task duration, but the average glance duration does not change with task time or glance frequency [109]. Drivers do not usually increase the glance duration for more difficult or longer tasks but rather increase the accumulated visual time sharing duration by increasing the number of glances away from the road [110]. As both single long glances and accumulated glance duration have been found to be detrimental for safety [110,111,112], a driver distraction detection algorithm based on visual behaviour should take both glance duration and repeated glances into account [113].

One one hand, high-resolution cameras placed throughout the cabin are needed to view the driver’s eyes from all head positions and at all times. Several economic and technical challenges of integrating and calibrating multiple cameras should be tackled to achieve this. Technically, eye orientation cannot always be measured in vehicular environments because eye region can be occluded by (1) sunlight reflections on eyeglasses; (2) the eye blink of the driver; (3) a large head rotation; (4) sunglasses; (5) wearing some kind of mascaras; (6) direct sunlight; (7) hats, caps, scarves; or (8) varying real world illumination conditions.

On the other hand, many security systems do not require such detailed gaze direction but they need coarse gaze direction to reduce false warnings [114,115]. For example, forward collision warning (FCW) systems need not only exterior observations but interior observations of the driver’s attention as well to reduce false warnings (distracting and bothering the driver), that is, coarse gaze direction can be used in order to control the timing of warning emission when the system detects that the driver is not facing forwards.

Taking into account that errors in facial feature detection greatly affect gaze estimation [116], many researchers have measured coarse gaze direction by using only head orientation with the assumption that coarse gaze direction can be approximated by head orientation [117]. Head pose is a strong indicator of a driver’s field of view and his/her focus of attention [59]. It is intrinsically linked to visual gaze estimation, which is the ability to characterize the direction in which a person is looking [118]. However, it also should be noted that drivers use a time-sharing strategy when engaged in a visual-manual task where the gaze is constantly shifted between the secondary task and the driving scene for short intervals of time [119] and often position the head in between the two involved gaze targets and only uses the eyes to quickly move between the two targets. In this situation, a face tracking algorithm would recognize this as a distracted situation based on head position, but the driver is constantly looking the road ahead. Therefore, in an ideal situation, both driver gaze tracking and eyes-off-road should be detected together [49].

**Figure 4:**
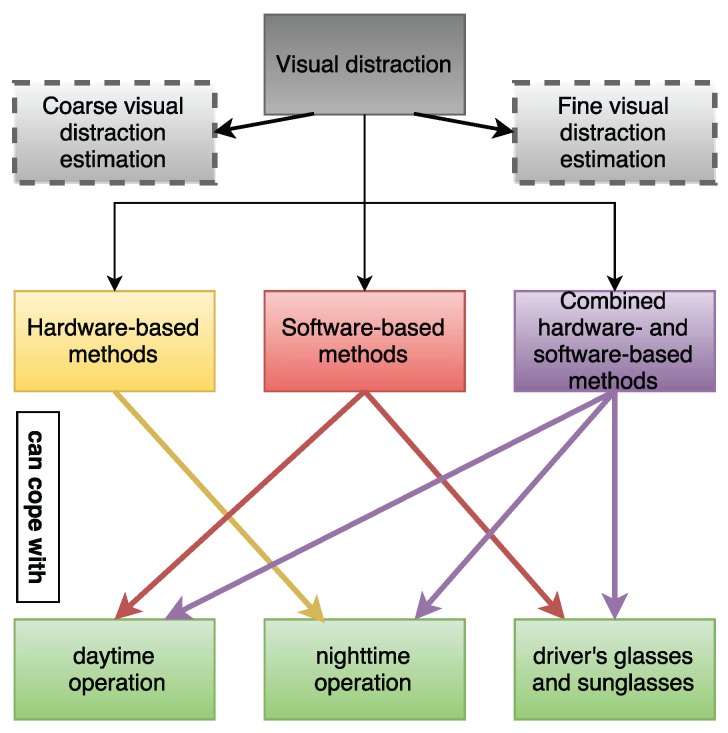
Visual distraction algorithms categorization.

In short, visual distraction can be categorized into two main approaches as it can be seen in Figure 4. In the first approach, which can be called “coarse”, researchers measured the coarse gaze direction and the focus of attention by using only head orientation with the assumption that the coarse gaze direction can be approximated by the head orientation. In the second approach, which can be called “fine”, researchers considered both head and eye orientation in order to estimate detailed and local gaze direction.

Moreover, considering its operating principles, visual distraction systems can be grouped in two main categories: hardware- and software-based methods. Additionally, some systems can combine these two approaches and therefore, a third category can also be considered, as seen in Figure 4.

### 4.1. Hardware-Based Methods to Extract Gaze Direction

Hardware-based approaches to head pose and gaze estimation rely on Near Infrared (NIR) illuminators to generate the bright pupil effect. These methods use two ring-type IR light-emitting diodes: one located near the camera’s optical axis and the other located far from it [120,121,122,123,124,125,126]. The light source near the camera optical axis makes a bright pupil image caused by the red-eye effect, and the other light source makes a normal dark pupil image. The pupil was, then, easily localized by using the difference between bright and dark pupil images. Ji et al. used the size, shape, and intensity of pupils, as well as the distance between the left and right pupil, to estimate the head orientation. Specifically, the authors used the pupil-glint displacement to estimate nine discrete gaze zones [121,122], a geometric disposition of the IR LEDs similar to that of Morimoto et al. [120] and two Charge Coupled Device (CCD) cameras embedded on the dashboard of the vehicle. In connection with the CCD cameras, the first one is a narrow angle camera, focusing on the driver’s eyes to monitor eyelid movement while the second one is a wide angle camera focusing on his/her head to track and monitor head movement. Based on this work, Gu et al. [124] proposed a combination of the Kalman filtering with the head motion to predict the features localization and used Gabor wavelet in order to detect the eyes constrained to the vicinity of predicted location. Another existent approach proposed by Batista et al. used dual Purkinje images to estimate a driver’s discrete gaze direction [125]. A rough estimation of the head-eye gaze was described based on the position of the pupils. The shape of the face is modeled with an ellipse and the 3D face pose is recovered from a single image assuming a ratio of the major and minor axes obtained through anthropometric face statistics. In this method, further research is necessary in order to improve the accuracy of the face orientation estimation, which is highly dependent on the image face ellipse detection.

The aforementioned NIR illumination systems work particularly well at night. The major advantage of these methods is the exact and rapid localization of the pupil. However, performance can drop dramatically due to the contamination introduced by external light sources [126,127]. In addition, during daytime, sunlight is usually far stronger than NIR light sources and hence, the red-eye effect may not occur. Moreover, these methods could not work with drivers wearing glasses because the lenses create large specular reflections and scatter NIR illumination [127,128,129]. While the contamination due to artificial lights can easily be filtered with a narrow band pass filter, sunlight contamination will still exist [126]. Furthermore, such systems are vulnerable to eye occlusion caused by head rotation and blinking [114].

### 4.2. Software-Based Methods to Extract Gaze Direction

Combining facial feature locations with statistical elliptical face modelling, Batista et al. [83] presented a framework to determine the gaze of a driver. To determine the gaze of the face, an elliptical face modelling was used taking the eye’s pupil locations to constraint the shape, size and location of the ellipse. The proposed solution can measure yaw head rotation over [−30°, +30°] interval and pitch head rotation over [−20°, +20°] interval.

Furthermore, despite the technical challenges of integrating multiple cameras, Bergasa et al. [130] proposed a a subspace-based tracker based on head pose estimation using two cameras. More specifically, the initialization phase was performed using the Viola and Jones algorithm [40] and a 3D model of the face was constructed and tracked. In this work, head pose algorithm, which was the base for visual distraction estimation, could track the face correctly up to [−40°, +40°].

A limitation of the software-based methods is the fact that they cannot often be applied at night [126,131]. This has motivated some researchers to use active illumination based on IR LEDs, exploiting the bright pupil effect, which constitutes the basis of these systems [126,131] (explained in previous section), or combine both methods, which can be seen in the next section.

### 4.3. Hardware- and Software-Based Methods to Extract Gaze Direction

Lee et al. [114] proposed a system for both day and night conditions. A vision-based real-time gaze zone estimator based on a driver’s head orientation composed of yaw and pitch is proposed. The authors focused on estimating a driver’s gaze zone on the basis of his/her head orientation, which is essential in determining a driver’s inattention level. For night conditions, additional illumination to capture the driver’s facial image was provided. The face detection rate was higher than 99% for both daytime and nightime.

The use of face salient points to track the head was introduced by Jimenez et al. [132], instead of attempting to directly find the eyes using object recognition methods or the analysis of image intensities around the eyes. The camera was modified to include an 850 nm band-pass filter lens covering both the image sensor and the IR LEDs in order: (a) to improve the rejection of external sources of IR radiation and reduce changes in illumination and (b) to facilitate the detection of the pupils, because the retina is highly reflective of the NIR illumination of the LEDs. An advantage of salient points tracking is that the approach is more robust to the eyes occlusion whenever they occur, due to the driver’s head or body motion.

Later on, the same authors extended their prior work in order to improve non-invasive systems for sensing a driver’s state of alert [133]. They used a kinematic model of the driver’s motion and a grid of salient points tracked using the Lukas-Kanade optical flow method [132]. The advantage of this approach is that it does not require one to directly detect the eyes, and therefore, if the eyes are occluded or not visible from the camera when the head turns, the system does not loose the tracking of the eyes or the face, because it relies on the grid of salient points and the knowledge of the driver’s motion model. Experiments involving fifteen people showed the effectiveness of the approach with a correct eyes detection rate of 99.41% on average. It should be noted that this work is focused on sensing the drivers’ state of alert, which is calculated measuring the percentage of eyelid closure over time (PERCLOS), and it is not focused on distraction detection.

Eyes Off the Road (EOR) detection system is proposed in [49]. The system collects videos from a CCD camera installed on the steering wheel column and tracks facial features. Using a 3D head model, the system estimates the head pose and gaze direction. For night time operation, the system requires an IR illumination. The proposed system does not suffer from the common drawbacks of NIR based systems [121,122,125], because it does not rely on the bright pupil effect. The system works reliably with drivers of different ethnicities wearing different types of glasses. However, if the driver is wearing sunglasses, it is not possible to robustly detect the pupil. Thus, to produce a reliable EOR estimation in this situation, only head pose angles are taken into account.

Cyganek et al. [134] proposed a setup of two cameras operating in the visible and near infra-red spectra for monitoring inattention. In each case (visible and IR) two cascade of classifiers are used. The first one is used for the detection of the eye regions and the other for the verification stage.

Murphy-Chutorian et al. used Local Gradient Orientation (LGO) and Support Vector Regression (SVR) to estimate the driver’s continuous yaw and pitch [135]. They used head pose information extracted from a LGO and SVR to recognize drivers’ awareness. The algorithm was further developed in [59] by introducing a head tracking module built upon 3D motion estimation and a mesh model of the driver’s head. There is a general weakness here as the tracking module may easily diverge from face shapes that are highly different to the given mesh model.

### 4.4. Driver Distraction Algorithms Based on Gaze Direction

In these previous Section 4.1, Section 4.2 and Section 4.3, gaze direction is extracted using different methods. The next step is to detect distraction using gaze direction regardless of the type of method used to extract this information, and hence, is commented as follows.

Many software-based methods have been proposed in order to detect visual distraction, many of which, rely on “course” information extracted from visual cues [114,136,137,138,139]. Hattori et al. [136] introduced a FCW system using drivers’ behavioural information. Their system determines distraction when it detects that the driver is not looking straight ahead. Following this approximation, an Android app [137] has been developed to detect and alert drivers of dangerous driving conditions and behaviour. Images from the front camera of the mobile phone are scanned to find the relative position of the driver’s face. By means of a trained model [38] four face related categories were detected: (1) no face is present; (2) facing forwards, towards the road; (3) facing to the left and (4) facing to the right. Another related system is proposed by Flores et al. [138] where, in order to detect distraction, if the system detects that the face position is not frontal, an alarm cue is issued to alert the driver of a danger situation. Lee et al. [114] proposed a vision-based real-time gaze zone estimator based on a driver’s head orientation composed of yaw and pitch. This algorithm is based on normalized histograms of horizontal and vertical edge projections combined with an ellipsoidal face model and a SVM classifier for gaze estimation. In the same research line but in a more elaborated fashion, Yuging et al. [139] used machine vision techniques to monitor the driver’s state. The face detection algorithm is based on detection of facial parts. Afterwards, the facial rotation angle is calculated based on the analysis of the driver’s head rotation angles. When the angle of facial orientation is not in a reasonable range and lasts for a relatively long time, it can be thought that the driver is distracted and warning information will be provided.

Additionally, other software-based approaches rely on “fine” information considering both head and eye orientation in order to estimate distraction [83,130,140,141]. Pohl et al. [140] focused on estimating the driver’s visual distraction level using head pose and eye gaze information with the assumption that the visual distraction level is non-linear: visual distraction increased with time (the driver looked away from the road scene) but nearly instantaneously decreased (the driver re-focused on the road scene). Based on the pose and eye signals, they established their algorithm for visual distraction detection. Firstly, they used a Distraction Calculation (DC) to compute the instantaneous distraction level. Secondly, a Distraction Decision-Maker (DDM) determined whether the current distraction level represented a potentially distracted driver. However, to increase the robustness of the method, also the robustness of the eye and head tracking device to adverse lighting conditions has to be improved.

Bergasa et al. [126] presented a hardware- and software-based approach for monitoring driver vigilance. It is based on a hardware system, for real time acquisition of driver’s images using an active IR illuminator and a software implementation for real time pupil tracking, ocular measures and face pose estimation is proposed. Finally, driver’s vigilance level is determined from the fusion of the measured parameters into a fuzzy system. The authors yielded an accuracy percentage close to 100% both at night and for users not wearing glasses. However, the performance of the system decreases during daytime, especially in bright days, and at the moment, the system does not work with drivers wearing glasses [126].

Recently, Lee et al. [141] evaluated four different vision-based algorithms for distraction under different driving conditions. These algorithms were chosen for their ability to distinguish between distracted and non-distracted states using eye-tracking data [141]. The resulting four algorithms, summarized in Table 5, are commented next:

1. Eyes off forward roadway (EOFR) estimates distraction based on the cumulative glances away from the road within a 6-s window [7].
2. Risky Visual Scanning Pattern (RVSP) estimates distraction by combining the current glance and the cumulative glance durations [142].
3. “AttenD” estimates distraction associated with three categories of glances (glances to the forward roadway, glances necessary for safe driving (i.e., at the speedometer or mirrors), and glances not related to driving), and it uses a buffer to represent the amount of road information the driver possesses [143,144,145].
4. Multi distraction detection (MDD) estimates both visual distraction using the percent of glances to the middle of the road and long glances away from the road, and cognitive distraction by means of the concentration of the gaze on the middle of the road. The implemented algorithm was modified from Victor et al. [146] to include additional sensor inputs (head and seat sensors) and adjust the thresholds for the algorithm variables to improve robustness with potential loss of tracking.

Considering the results of the ROC curves, AUC values, accuracy and precision, it is apparent that a trade-off exists between ensuring distraction detection and avoiding false alarms, which complicates determining the most promising algorithm. More specifically, the MDD algorithm showed the best performance across all evaluation metrics (accuracy, precision, AUC). Although the EOFR algorithm had promising AUC values, the AttenD algorithm often yielded better accuracy and precision. Additionally, the RVSP algorithm consistently yielded the lowest values for both accuracy and precision, but yielded a slightly higher AUC value than AttenD. All of the algorithms succeeded in detecting distraction well above chance detection (AUC = 0.5). The performance of the algorithms varied by task, with little difference in performance for the looking and reaching task (bug) but more stark differences for the looking and touching (arrows). The AUC for each task for each algorithm is provided in Table 5.

## 5. Cognitive Distraction

**Figure 5:**
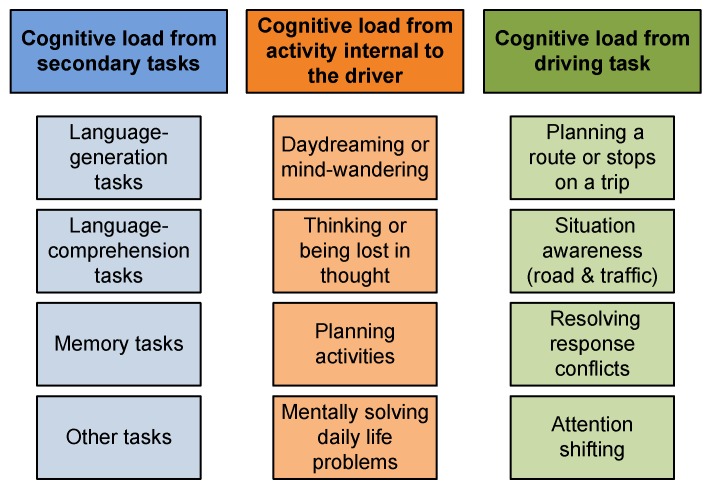
Classification of main types and subtypes of cognitive load while driving.

Cognitive distraction is a critical area of concern with regard to driver distraction, particularly as related to tasks of listening and conversing, but also, as related to spontaneously occurring processes like daydreaming or becoming lost in thought, which may occur really often on long drives. The term “cognitive load” can be defined as any workload imposed on a driver’s cognitive processes [26]. There are several types (and subtypes) of scenarios where cognitive load may occur during (see Figure 5), and therefore, affect driving. For further information, the reader may refer to [26]. These include:

1. Cognitive load imposed by secondary tasks undertaken while driving.
2. Cognitive load associated with the driver’s internal activity.
3. Cognitive load arising from the driving task itself.

### 5.1. Behavioral and Physiological Indicators of Cognitive Load

The research literature documents several types of measures associated with periods of cognitive load. Secondary tasks imposing cognitive load lead to: (1) a high percentage of glances on the forward road and; (2) unusually long glances on the forward road. These two metrics together have been found to be uniquely indicative of cognitive loads [110,147]. Moreover, a narrowing of the spatial extent of scanning [147] is also produced, which is reflected in slightly fewer glances to locations where the mirrors, the speedometer and the areas peripheral to the road centre are located [26,148]. As a result, cognitive load may cause an increasing gaze concentration towards the middle of the road [11].

An eye-gaze pattern could be used to differentiate the action of only driving from driving under the influence of any cognitive task [147]. Drivers under cognitive distraction had fewer saccades per unit time, which was consistent with less exploration of the driving environment [149]. Saccades may be a valuable index of mental workload [150]. In fact, the standard deviations of both eye and head movement could be suitable for detecting cognitive distraction causing gaze concentration and slow saccades when drivers are looking forward [151]. A higher blink rate and a shrink in visual searching range were observed when the driver was cognitively distracted [152]. Kircher et al. [144] indicated the percentage of time the driver spent observing the road ahead, which is called the percentage road center (PRC) of gaze direction, was more than 92% under cognitive distraction in a field study.

Therefore, both glance and blink measures can be used to detect cognitive distraction. For example, He et al. [153] have observed that mind-wandering has effects on glance patterns and blink rates similar to those observed in periods of cognitive secondary task load. During mind-wandering, there is also an increasing concentration of gaze on the forward road with concomitant narrowing of scanning, longer glances on the forward road, and changes in blink rate [26]. Results from [154] suggested that performance data and oculomotor scanning behavior may allow the detection of drivers’ mind wandering episodes before they are recognized by the driver himself/herself, potentially providing interventions to detect inattentiveness and alert drivers. Blink rate seems to be a promising indicator of cognitive processing [27]. However, there are measurement issues that may affect how successfully it can be applied in discriminating different types of task loading during driving. For example, there are some questions left about whether it offers sufficient sensitivity when extracted from real world data acquired from a complex task like driving wherein there are inherent temporal variations in driving task load [26,27].

Physiological measures can also be used to detect cognitive load. The average value of pupil diameter is suitably used as a physiological feature for detection of cognitive distraction [155]. When cognitive loads such as arithmetic or conversation were imposed to the subjects, dilation of pupils occurred by acceleration of the sympathetic nerve, resulting in an increase of diameter of pupils [156]. The average value of pupil diameter caused by cognitive loads, such as arithmetic, increased by 13.1% compared with ordinary driving [156]. The tests were performed in a driver simulator in controlled settings. Further experiments are required in a naturalistic setting. Moreover, additional works highlighted the difficulty in estimating cognitive load using pupil diameter during a dialogue task [157] or in different lighting conditions [158].

The same limitation applies to other physiological measures, as Heart Rate (HR), which tends to increase as cognitive task load raises [159]. The traditional method to quantify these physiological measures is by wearing physiological sensors. However, HR measurements can be acquired using computer vision techniques, and consequently, special care has been taken reviewing HR information. Additionally, it is considered a good indicator of fatigue, stress and cognitive load.

By means of the use of HR information the cognitive state of a driver can be monitored [160] in controlled settings. Changes in HR have been noted during certain driving tasks [161]. Similarly, Apparies et al. [162] showed that HR and Heart Rate Variability (HRV) may serve as early indicators of fatigue. In general, HRV specifically measures mental workload, while HR measures physical one [163]. HRV analysis is a strong indicator of mental stress or workload caused by driving tasks [162,164,165]. Experiments carried out in a driving simulator by Zhao et al. [166] found that human heart rates violently fluctuate during a mental stress situation. Ostlund et al. [167] and Miller et al. [165] identified both HR and HRV as promising indicators of the driver’s stress level, by increasing HR and decreasing HRV [165,167]. Physiological measures, such as HR and skin conductance level, tend to increase as cognitive task load increases [159].

There are some research works able to extract HR and HRV from face video images in real time from human faces [168,169,170]. Eulerian Video Magnification framework [171] can be also used to obtain human pulse from a video sequence [172]. In [173], the authors described an approach offering a non-invasive, non-contact means of cardiac monitoring. Once the HRV time series are extracted, feature generation, feature selection and classification should be performed. The conventional method that uses Fast Fourier Transform (FFT) analysis on HRV is 2-min long. In [174], a new method developed by using wavelet-based feature and SVM for classification uses only 1-min HRV signals. Moreover, this method increases accuracy, sensitivity and specificity compared to FFT-based results.

Therefore, concerning cardiovascular measures, they have been reported to be sensitive to mental workload changes and both HR and HRV are widely adopted mental workload measures because they are easy to use and provide fundamental information about the autonomic nervous system [175]. Most methods [168,169,170] enable low-cost, non-contact cardiovascular activity monitoring using regular RGB cameras by analyzing minute skin color changes caused by periodic blood flow. Nevertheless, for automotive applications, these methods can encounter difficulties under different illumination conditions [176]. In [176], the authors proposed an artifact reduction method, which is caused by lighting variation. Another option is to use an IR-based camera system suitable for automotive applications [177].

To conclude this section, the use of physiological parameters can be used to monitor the cognitive state of the driver. Many of these parameters have been described in controlled settings, but further experiments are required to validate their capability in naturalistic conditions. The main algorithms in this matter are included in the next section.

### 5.2. Algorithms

Zhang et al. [178] used a decision tree approach to estimate drivers’ cognitive workload from eye gaze-related features and driving performance. Liang, Reyes, et al. [179] showed that the SVM models can also detect cognitive distraction. The model’s accuracy and sensitivity increased with window size, suggesting that using longer periods to summarize the data made the distraction signal easier for the models to detect. The conclusion was that the best models were obtained using 40-s window size. Additionally, Liang, Lee, et al. [180] also used Bayesian Network (BN) models and found that they could identify cognitive load reliably for simulator data, and also found that Dynamic Bayesian Networks (DBNs), which considered time dependencies of driver’s behaviour, gave a better performance than static BN models. This fact suggests that time-dependent relationship is critical in estimating the cognitive state of the driver. However, to train DBN models, longer training sequences are necessary to obtain more accurate and sensitive models. The results obtained in [180] using BNs, which stated that window size did not affect model performance, clearly conflict with those of Liang et al. [179], which found that larger window sizes improved the detection of cognitive distraction, although another data mining method, SVM, was applied in that study. An additional work from Liang et al. [181] compared SVMs, SBNs, and DBNs in detecting cognitive distraction using the best parameter settings from the same dataset used in the previous two studies [179,180]. DBNs produced the most accurate and sensitive models compared to SBN and SVM. Based on the comparisons of SVMs and BNs, Liang et al. [27,182,183] used a hierarchical layered algorithm, which incorporated both a DBN and a supervised clustering algorithm, to identify feature behaviors when drivers were in different cognitive states. This layered algorithm includes a DBN algorithm at the higher level to model the time-dependent relationship of driver behavior and a supervised clustering algorithm at the lower level to identify feature behaviors. The layered algorithm overcomes the disadvantages of DBNs and significantly improves computational efficiency in training and prediction. Miyaji et al. [184] proposed an approach to detect eye and head movement tracked via standard deviation and categorized features for pattern recognition by using AdaBoost method to detect distraction. The authors compared the performance achieved by both SVM and AdaBoost in estimating cognitive workload, finding that AdaBoost could achieve higher accuracy. Additionally, Miyaji et al. [156] introduced a mixed method by applying a SVM and an AdaBoost classifier for three parameters: (1) heart rate; (2) visual information (standard deviation of both gaze and head rotation angles) and (3) pupil diameter to assess the level of the driver’s vigilance. Recently, a new machine learning tool, Extreme Learning Machine (ELM) [185,186]), has gained much attention due to its simple structure, high generalization capability, and fast computational speed. For example, in [187], ELM and SVM were applied to detect drivers’ workload using eye movement, as well as eye movement combined with driving performance data. The results suggested that both methods can detect drivers’ workload at high accuracy, but ELM outperformed SVM in most cases.

The results of all the works mentioned so far can be summarized in Table 6. Common features include the use of eye gaze-related features, driving performance, pupil diameter features and HR. It should also be noted that very good results can be obtained using only eye gaze-related features. Additionally, many supervised machine learning techniques have been proposed so far: decision trees, SVM, BN, DBN, AdaBoost or ELM.

All these distraction detection systems are based on supervised learning, meaning that the training of such systems need to be “supervised” by human experts by providing a target set for training data containing distraction status. The supervised learning paradigm is only suitable for early stage research and may not be suitable for implementation in real driving cases because of the huge cost and difficulty of creating a target distraction status set, which would require additional subjective ratings by the driver [115], post-processing by the experimentalists [56], or additional computation based on data from other sources [179]. For example, in a recent study [188], labeling drivers’ distraction state involves the development of Graphical User Interface (GUI), the training of external evaluators, and the actual labeling time, which is approximately 21.5 h of manpower (43 min per one of the 30 evaluator) to label the entire video segments. For naturalistic driving, where the driver voluntarily decides which tasks to perform at any time, the labeling process can become infeasible. On the other hand, data without known distraction states (unlabeled data) can be collected readily, e.g., from drivers’ naturalistic driving records.

With the purpose of tackle these deficiencies, Unsupervised and Semi-Supervised algorithms can be used. For example, in [12], Semi-Supervised Extreme Learning Machine (SS-ELM) is proposed for drivers’ distraction detection. SS-ELM outperformed supervised ELM in both accuracy and model sensitivity, suggesting that the proposed semi-supervised detection system can extract information from unlabeled data effectively to improve the performance. SS-ELM based detection system has the potential of improving accuracy and alleviating the cost of adapting distraction detection systems to new drivers, and thus, more promising for real world applications. However, several points are unclear from these preliminary results [12] further explored in [189], where the Semi-Supervised Learning (SSL) paradigm is introduced to real time detection of distraction based on eye and head movements.

In [189], two graph-based SSL methods were compared with supervised learning methods. These algorithms are detailed as follows. Laplacian Support Vector Machine (LapSVM), which is an extension of SVMs to SSL under manifold regularization framework [190], and SS-ELM were compared with three supervised learning methods (static BN with Supervised Clustering (SBN-SC) [180,183], ELM and SVM) and one low-density-separation-based method (Transductive SVM (TSVM) [191]). To capture realistic eye and head movements patterns, data from an on-road experiment were used. By utilizing unlabeled data, the graph-based semi-supervised methods reduced the labeling cost and improved the detection accuracy. The highest accuracy of 97.2% and G-mean of 0.959 were achieved by SS-ELM. The benefits of using SSL methods increased with the size of unlabeled data set showing that by exploring the data structure without actually labeling them, extra information to improve models performance can be obtained.

It is worth noting that cognitive distraction detection is only performed in “laboratory settings” and not in real conditions. In real life situations, when the driver is under cognitive load (e.g., mind wandering): (1) he is alone and does not interact with anybody; (2) he is also the only one who can decide whether or not to activate the attentional processing of distractive thoughts [192]; and (3) drivers are likely to be performing multiple tasks at the same time (e.g., talking on the mobile phone and listening to music). Moreover, there are two main limitations intrinsic to laboratory-based studies. First of all, most of these studies require that the execution of predefined tasks last for no more than some minutes. In our opinion, such experiments make it very difficult, if not impossible, to infer, for instance, the long-term effectiveness of for example, warning signals, monotonous driving (in general, real driving), on the basis of the results of experiments that are typically so short; And secondly, the drivers are abnormally vigilant to the driving task because they are being observed [193]. In connection with this point, the use of physiological parameters, which form the basis for cognitive distraction detection, have also been extracted in controlled settings and not in real conditions.

## 6. Mixing Types of Distraction

There are some algorithms trying to detect mixing types of distraction, whose results can be seen in Table 7. In [194], facial features are extracted to detect both visual and cognitive distractions. Binary classifiers (normal vs distracted) are built for visual and cognitive distraction detection. Gaze and Action Units (AU) features are useful in order to detect visual distractions, while AU features are particularly important for cognitive distractions. It should be pointed out that the cognitive tasks considered in this study are closely related to talking activities.

Liu et al. [196] applied Cluster Regularized Extreme Learning Machine (CR-ELM) for detecting mixing types of distraction. Compared with the traditional ELM, CR-ELM introduces an additional regularization term penalizing large covariance of training data within the same clusters in the output space. CR-ELM, ELM and SVM were compared to predict mixing types of distraction. They simulated the mixing types of distraction by combining two types of distracting driving activities (a visual task and a cognitive one). CR-ELM showed lower error rate on most of the 11 subjects (see Table 7).

There are other approaches trying to merge both RGB and depth images to get the features to be used by the algorithms [195,197]. Craye et al. [195] extracted features from face and body using both color and depth images in order to build a distraction system, which is composed of four sub-modules: eye behaviour (gaze and blinking), arm position, head orientation and facial expressions. The information from these modules are merged together using two different classification techniques: Adaboost classifier and Hidden Markov Model (HMM). A set of video sequences was collected to test the system. Five distractive tasks were recorded and manually labelled for training and evaluation. HMM outperforms Adaboost for most drivers. Finally, a vision-based driver distraction is investigated using several machine learning techniques in [197]. IR and Kinect cameras were used in this system, where five visual cues were calculated: arm position, eye closure, eye gaze, facial expressions and head orientation. These cues were fed into a classifier, such as Adaboost, HMM, RF, SVM, Conditional Random Field (CRF) or NN, in order to detect and recognize the type of distraction.

## 7. The Relationship between Facial Expressions and Distraction

Facial expressions can be described at different levels [198]. A widely used description is Facial Action Coding System (FACS) [199], which is a human-observer-based system developed to capture subtle changes in facial expressions. With FACS, these expressions are decomposed into one or more AUs [200]. AU recognition and detection have attracted much attention recently [201]. Meanwhile, psychophysical studies indicate that basic emotions have corresponding universal facial expressions across all cultures [202]. This is reflected by most current facial expression recognition systems attempting to recognize a set of prototypic emotional expressions including disgust, fear, joy, surprise, sadness and anger [201], which can be helpful in predicting driving behaviour [203].

Therefore, in this work, main facial expression works in the driving environment are described in accordance with the two aforementioned levels (FACS and prototypic emotional expressions) and how they are related with distraction.

On one hand, in connection with FACS and distraction while driving, the reference work is the one proposed by Li et al. [194]. The authors performed the analysis of driver’s facial features under cognitive and visual distractions. In addition to the obvious facial movement associated with secondary tasks such as talking, they hypothesized that facial expression can play an important role in cognitive distraction detection. They studied the top five features (from a total of 186 features) to predict both cognitive and visual distraction. For cognitive distraction, the most important features to consider are: (1) head yaw; (2) Lip Corner Depressor (AU15); (3) Lip Puckerer (AU18); (4) Lip Tightener (AU23) and (5) head roll. For visual distraction, the most important features to consider are: (1) Lip Tightener (AU23); (2) jaw drop (AU26); (3) head yaw; (4) Lip Suck (AU28) and (5) Blink (AU45). The results indicated that gaze and AU features are useful for detecting visual distractions, while AU features are particularly important for cognitive distractions. It should be pointed out that since the cognitive tasks considered in this study are closely related to talking activities, their future work will include the analysis of other cognitive tasks (e.g., thinking or solving math problems).

On the other hand, in connection with prototypic emotional expressions, there are some works trying to study how these emotions affect behaviour.

The relationship between emotion and cognition is complex, but it is widely accepted that human performance is altered when a person is in any emotional state. It is really important to fully understand the impact of emotion on driving performance because, for example, roadways are lined with billboard advertisements and messages containing a lot of different emotional information. Moreover, the distracting effects of emotions may come in other forms, such as cell phone, passenger conversations, radio information or texting information [204]. For example, Chan et al. [204] conducted a study to examine the potential for distraction from emotional information presented on roadside billboards. The findings in this study showed that emotional distraction: (a) can seriously modulate attention and decision-making abilities and have adverse impacts on driving behavior for several reasons and (b) can impact driving performance by reorienting attention away from the primary driving task towards the emotional content and negatively influence the decision-making process. In another study with a similar line of work, Chan et al. [205] showed that emotion-related auditory distraction can modulate attention to differentially influence driving performance. Specifically, negative distractions reduced lateral control and slowed driving speeds compared to positive and neutral distractions.

Some studies have shown that drivers who are more likely to become angry (e.g., those with high trait anger rates) tend to engage in more aggressive behavior on the road, which can result in negative outcomes such as crashes [206]. Moreover, anger negatively influences several driving performance and risky behaviors such as infractions, lane deviations, speed, and collisions [207].

In conclusion, aggressiveness and anger are emotional states that extremely influence driving behaviour and increase the risk of accident. However, a too low level of activation (e.g., resulting from emotional states like sadness) also leads to reduced attention and distraction as well as prolonged reaction time and, therefore, lowers driving performance [208]. On this basis, research and experience have demonstrated that being in a good mood is the best precondition for safe driving and that happy drivers produce fewer accidents [209]. In other words, *happy drivers are better drivers* [208,210]. Facial expression and emotion recognition can be used in advanced car safety systems, which, on one hand, can identify hazardous emotional drivers’ states that can lead to distraction and, on the other, can provide tailored (according to each state and associated hazards) suggestions and warnings to the driver [211].

## 8. Sensors

Once the algorithms for distraction detection have been designed and implemented, the next step is to port them to an embedded device or system to be executed inside the car. However, porting a vision-based algorithm is not a straightforward step and some key factors should be taken into account. Furthermore, there is not a standard implementation platform, so different alternatives have been proposed by both the scientific community and the industry.

### 8.1. Porting a Vision Algorithm to an Embedded Automotive System

The implementation of computer vision applications in automotive environments is not straightforward because several requirements must be taken into account: reliability [212,213], real-time performance [213,214,215], low-cost [216,217,218,219], spatial constraints [217,219], low power consumption [220], flexibility [219], rapid prototyping [215,221], design requirements [217] and short time to market [217]. Therefore, there must be a trade-off among these design requisites [217]. Moreover, there is not a commonly accepted hardware and software platform, so different solutions have been proposed by the industry and the scientific community. Last but not least, some driver distraction guidelines and test procedures for all applications to be used while driving should be considered [222], and so should ADAs.

One approach can rely on the use of microprocessors, which incorporates the functions of a computer’s central processing unit (CPU) on a single integrated circuit (IC). For example, in [223], a vision-based system for monitoring the loss of attention, tested under day and night driving conditions, is proposed. The algorithm was cross-validated using brain signals and finally, implemented on a Single Board Computer (SBC). Another example is presented in [224], where a vehicle was equipped with a USB camera connected to the system in order to track the driver’s eyes for fatigue detection.

A similar approach is the use of digital signal processors (DSPs) [225], which can perform multiplications and additions in a single cycle and have parallel processing capabilities. DSPs have been used in image and audio signal processing when the use of microcontrollers was not enough. These processors were used in [215], where an optimized vision library approach for embedded systems was presented. VLIB is a software library that accelerates computer vision applications for high-performance embedded systems. By significantly speeding up pixel-intensive operations, the library provides more headroom for innovative algorithms, and enables processing of more channels at higher resolutions. Authors optimized the library for the Texas Instruments C64x/C64x+ DSP cores. Karuppusamy et al. [226] proposed an embedded implementation of facial landmarks detection based on both Viola-Jones face detector and facial landmarks detection using extended Active Shape Model (ASM) [227]. However, DSPs imply a much higher cost compared with other options such as field-programmable gate arrays (FPGAs) [228].

Another option is to use hardware implementation, since it can achieve a much better computational performance, where two types are commonly used namely, FPGA and ASIC. A FPGA is an integrated circuit designed to be configured by a customer or a designer after manufacture. FPGAs take advantage of high speed operations, especially for parallelizable operations achieving good performance in face monitoring applications [229,230,231,232]. For example, several well-known algorithms have been used and optimized for this field of application, such as: (a) spatial and temporal filtering, motion detection and optical flow analysis [229] or (b) gray scale projection, edge detection with Prewitt operator and complexity functions [230]. Additionally, the use of Application-Specific Integrated Circuits (ASIC), which is an IC customized for a particular use rather than intended for general-purpose use, has also been considered [233]. FPGAs have an important advantage over ASICs: they are reconfigurable, which gives them some of the flexibility of software. ASICs are only used for high volume manufacturing and long series due to higher initial engineering cost.

Developing the whole application in hardware is a cumbersome task, so hybrid solutions have appeared combining both software and hardware implementations. The work in [234] describes a System on a Chip (SOC) designed to support a family of vision algorithms. However, this system uses an ASIC, so it cannot be completely reconfigured. This important drawback makes impossible to update the device. A generic embedded hardware and software architecture was proposed to design and evaluate ADAS vision applications [221]. Although the system is useful to test some vision applications, the performance obtained in the case study showed that the system is not powerful enough to run more complex applications including I/Os management, vehicle communications or more demanding vision applications. In [219], a reconfigurable embedded vision system reaching the requirements of ADAS applications is presented. A SOC, which is formed by an FPGA with a dual core ARM, is prepared to be easily reconfigured. A lane departure warning system was implemented in the case study obtaining a good computational performance. The obtained computational time allows the system to include another more complex vision algorithm running in parallel. In [235], they proposed an approach to predict performances of image processing algorithms on different computing units of a given heterogeneous SOC.

Despite the fact that in recent years some authors have been trying to propose some architectures in order to achieve some key factors in embedded ADAS systems inside a vehicle [219,221,235], these efforts do not seem to be sufficient to reach the requirements stated before. The use of microprocessors in embedded computer vision-based systems has experienced a significant growth in recent years. Moreover, current premium cars implement more than 90 Electronic Control Units (ECU) with close to 1 Gigabyte embedded software code [236]. In 2018, 30% of the overall vehicle cost is predicted to stem from vehicle electronics [237]. The independence of different applications (with different criticality levels) running on the same platform must be made evident. Therefore, the development of embedded automotive systems has become quite complex. To that end, the use of standards and frameworks is indispensable.

### 8.2. Commercial Sensors

#### 8.2.1. Smart Eye

The Smart Eye [77] system is a well-suited head and gaze tracking method for the demanding environment of a vehicle cabin and flexible to cope with most research projects. It consists of a multi-camera system running on a single PC and on a single algorithm. The system is scalable from 2 up to 8 cameras allowing 360° head and eye tracking. A typical configuration inside a vehicle cabin is composed of four cameras with two IR lightings, located on the dashboard on either side of the steering wheel. Smart Eye offers a sampling rate of 60 Hz (up to 8 cameras) or 120 Hz (up to 4 cameras). The field of view, depending on the number of cameras, is in the range of 90°–360°. The data output includes over 145 values covering, among others, gaze, eyelid, pupilometry and head tracking, raw and filtered gaze, blinks, fixations and saccades. Smart Eye has been used in several driver assistance and inattention systems, such as [76,143,144,145,238].

#### 8.2.2. EyeAlert

EyeAlert [239], cited in several publications [128,240], has been conceived to detect driver inattention using computer vision and to generate a warning signal in case of dangerous situation. The EyeAlert system focuses entirely on the driver’s alertness levels or inattention to the road ahead, regardless of the time of the day or the weather conditions. Three models are available:

- EyeAlert EA410 detects both distracted and fatigue driving. The EA410 has a highly integrated IR camera, a computer, an image processing unit and an alarm. The EA410 technology is protected by over ten patents. The system will also respond in case the driver does not focus on driving.
- EyeAlert EA430 with GPS detects both distracted and fatigue driving. Moreover, a minimum speed threshold is programmed into the internal GPS to prevent false alarms in urban environments.
- EyeAlert EA450 with Data detects both distracted and fatigue driving. Additionally, minimum speed threshold, sensitivity, volume and data can be remotely programmed. The minimum speed and sensitivity controls allow the reduction of false alarms in highway and urban environments.

#### 8.2.3. Seeing Machines

Seeing Machines [241] builds image-processing technology that tracks the movement of a person’s eyes, face, head, and facial expressions. It monitors fatigue and distraction events in real-time and uses IR technology to provide fatigue and distraction monitoring at any time of the day. The system can also combine multiple camera sensors to detect a wider range of movements. The Seeing Machines’ system continuously measures operator eye and eyelid behaviour to determine the onset of fatigue and micro sleeps and delivers real-time detection and alerts.The system has been used in many different driver assistance and inattention systems [11,142,151,156,242,243,244].

#### 8.2.4. Visage Technologies AB

Visage Technologies AB [245] provides a state-of-the-art commercial head tracker based on feature-point detection and tracking of the nose boundary and eye regions. Visage SDK finds and tracks the face and facial features, including gaze direction, in video sequences in real time. It provides pupil coordinates, 3D gaze direction as well as (with a calibration step) screen-space gaze point. Visage Technologies also features support for embedded systems like FPGA and IR light tracking for poor lighting conditions.

#### 8.2.5. Delphi Electronics Driver Status Monitor

Delphi Electronics, a major automotive electronics manufacturer, developed a single camera Driver Status Monitor (DSM) [246]. By detecting and tracking the driver’s facial features, the system analyzes eye-closures and head pose to infer his/her fatigue or distraction. This information is used to warn the driver and to modulate the actions of other safety systems. The system includes the use of NIR illumination, an embedded processing unit, as well as the camera (resolution of 640 × 480 pixels).

#### 8.2.6. Tobii Technologies

Tobii Technologies develops Tobii’s eye-tracking technology for integration into volume products such as computers, computer games, virtual reality and cars. The Tobii platform consists of two-camera sensors, placed at different angles, and operating at IR frequencies to eliminate interference from external light. The system can distinguish whether the driver’s eyes are open or closed or if the driver has turned his/her head. The sensors work even when the driver is wearing glasses or sunglasses. By observing the specifics of eyelid closure, in combination with eye gaze patterns, an active safety system powered by Tobii’s eye tracking sensor can reliably detect if a driver is falling asleep and warns him/her properly. Moreover, Tobbi Technologies provides the Tobii EyeChip, which is a dedicated eye tracking SOC ASIC.

#### 8.2.7. SensoMotoric Instruments

SensoMotoric Instruments GmbH (SMI) [247] is a German company, whose eye tracking solutions can measure head position and orientation, gaze direction, eyelid opening, and pupil position and diameter. Eye trackers use a sampling rate of 120 Hz for head pose and gaze measurement, 120 Hz for eyelid closure and blink measurement, and 60 Hz for combined gaze, head pose, and eyelid measurement. It also provides PERCLOS information for drowsiness detection. It is a computer-based system and needs user calibration. In [248], SensoMotoric was used to recognize the pupil in each image in order to measure horizontal and vertical eye movements.

#### 8.2.8. Automobile Manufacturers

Nissan introduces its new Driver Attention Alert system with the 2016 Nissan Maxima, which was unveiled at the New York International Auto Show [249]. The automaker has highlighted that the new system will be able to detect drowsy and inattentive driving and it will alert the driver about the situation by giving visual and audible warning. Ford’s Driver Alert [250] seems only to detect drowsiness but not distraction. The Driver Alert system comprises a small forward-facing camera connected to an on-board computer. The camera is mounted on the back of the rear view mirror and is trained to identify lane markings on both sides of the vehicle. When the vehicle is on the move, the computer looks at the road ahead and predicts where the car should be positioned relative to the lane markings. Then, it measures where the vehicle actually is and, if the difference is significant, the system issues a warning. The Saab Driver Attention Warning System [251] detects visual inattention and drowsy driving. The system uses two miniature IR cameras integrated with Smart Eye technology [77] to accurately estimate head pose, gaze, and eyelid status. When a driver’s gaze is not located inside the primary attention zone (which covers the central part of the frontal windshield) for a predefined period, an alarm is triggered. Toyota has equipped their luxury Lexus models with their Driver Monitoring System [252]. The system permanently monitors the movement of the driver’s head when looking from side to side using a NIR camera installed at the top of the steering wheel column. The system is integrated into Toyota’s pre-crash system, which warns the driver when a collision is likely to happen. In 2007, Volvo Cars introduced Driver Alert Control to alert tired and non-concentrating drivers [253,254]. Based on the idea that the technology for monitoring eyes is not yet sufficiently mature and human behavior varies from one person to another, Volvo Cars developed the system based on car progress on the road. It is reported that Driver Alert Control monitors the car movements and assesses whether the vehicle is driven in a controlled or uncontrolled way. More recently, a Hyundai concept car (the Hyundai HCD-14) incorporates Tobbi Technologies to track the eyes [255].

## 9. Simulated vs. Real Environment to Test and Train Driving Monitoring Systems

The development of the computer vision algorithm only represents one part of all the cycle of the product design. One of the hardest tasks is to validate the whole system with the wide variety of driving scenarios [256]. In order to complete the whole “process development” of the vision-based ADAS, some key points are presented.

In order to monitor both the driver and his/her driving behaviour, several hardware and software algorithms are being developed, but they are tested mostly in simulated environments instead of in real driving ones. This is due to the danger of testing inattention in real driving environments [21]. Experimental control, efficiency, safety, and ease of data collection are the main advantages of using simulators [257,258]. Some researches have validated that driving simulators can create driving environment relatively similar to road experiments [259,260,261]. However, some considerations should be taken into account since simulators can produce inconsistent, contradictory and conflicting results. For example, low-fidelity simulators may evoke unrealistic driving behavior and, therefore, produce invalid research outcomes. One common issue is that real danger and the real consequences of actions do not occur in a driving simulator, giving rise to a false sense of safety, responsibility, or competence [262]. Moreover, simulator sickness symptoms may undermine training effectiveness and negatively affect the usability of simulators [262].

A study on distraction in both simulated and real environment was conducted in [11] and it was found out that the driver’s physiological activity showed significant difference. Engstorm et al. [11] stated that physiological workload and steering activity were both higher under real driving conditions compared to simulated environments. In [257], the authors compared the impact of a narrower lane using both a simulator and real data, showing that the speed was higher in the simulated roads, consistent with other studies. In [263], controlled driving yielded more frequent and longer eye glances than the simulated driving setting, while driving errors were more common in simulated driving. In [167], the driver’s heart rate changed significantly while performing the visual task in real-word driving relative to a baseline condition, suggesting that visual task performance in real driving was more stressful.

After the system is properly validated in a driver simulator, it should be validated in real conditions as well, because various factors including light variations and noise can also affect the driver’s attention. The application on a real moving vehicle presents new challenges like changing backgrounds and sudden variations of lighting [264]. Moreover, a useful system should guarantee real time performance and quick adaptability to a variable set of users and to natural movements performed during driving [264]. Thus, it is necessary to make simulated environments appear more realistic [203].

To conclude, in most previous studies, independent evaluations using different equipment and conditions (mainly simulated environments) resulted in time-consuming and redundant efforts. Moreover, inconsistency in the algorithm performance metrics makes it difficult to compare algorithms. Hence, the only way to compare most algorithms and systems is the metrics provided by each author when comparing their values, but with scarce information about the used images and conditions. Public data sets covering simulated and real driving environments should be released in the near future, as stated by some authors previously [203].

## 10. Privacy Issues Related to Camera Sensors

Although there is a widespread agreement for intelligent vehicles to improve safety, the study of driver behaviour to design and evaluate intelligent vehicles requires large amounts of naturalistic driving data [265]. However, in current literature, there is a lack of publicly available naturalistic driving data largely due to concerns over individual privacy. It also should be noted that a real-time visual-based distraction detection system does not have to save the video stream. Therefore, privacy issues are mostly relevant in research works were video feed is collected and stored to be studied at a later stage, for example in the large naturalistic studies conducted in the US.

Typical protection of the individuals’ privacy in a video sequence is commonly referred as “de-identification” [266]. Although this fact will help protect the identities of individual drivers, it impedes the purpose of sensorizing vehicles to control both drivers and their behaviour. In an ideal situation, a de-identification algorithm would protect the identity of drivers while preserving sufficient details to infer their behaviour (e.g., eye gaze, head pose or hand activity) [265].

Martin et al. [265,267] proposed the use of de-identification filters to protect the privacy of drivers while preserving sufficient details to infer their behaviour. Following this idea, a de-identification filter preserving only the mouth region can be used for monitoring yawning or talking and a de-identification filter preserving eye regions can be used for detecting fatigue or gaze direction, which is precisely proposed by Martin et al. [265,267]. More specifically, the authors implemented and compared de-identification filters made up of a combination of preserving eye regions for fine gaze estimation, superimposing head pose encoded face masks for providing spatial context and replacing background with black pixels for ensuring privacy protection. A two-part study revealed that human facial recognition experiment had a success rate well below the chance while gaze zone estimation accuracy disclosed 65%, 71% and 85% for One-Eye, Two-Eyes and Mask with Two-Eyes, respectively.

Fernando et al. [268] proposed to use video de-identification in the automobile environment using personalized Facial Action Transfer (FAT), which has recently attracted a lot of attention in computer vision due to its diverse applications in the movie industry, computer games, and privacy protection. The goal of FAT is to “clone” the facial actions from the videos of a person (source) to another one (target) following a two-step approach. In the first step, their method transfers the shape of the source person to the target subject using the triangle-based deformation transfer method.In the second step, it generates the appearance of the target person using a personalized mapping from shape changes to appearance changes. In this approach video de-identification is used to pursue two objectives: (1) to remove person-specific facial features and (2) to preserve head pose, gaze and facial expression.

## 11. General Discussion and Challenges Ahead

The main visual-based approaches reviewed in this paper are summarized in Table 8 according to some key factors.

A major finding emerging from two recent research works reveals that just-driving baselines may, in fact, not be “just driving” [26,269], containing a considerable amount of cognitive activity in the form of daydreaming and lost-in-thought activity. Moreover, eye-gaze patterns are somewhat idiosyncratic when visual scanning is disrupted by cognitive workload [27]. Additionally, “look-but-failed-to-see” impairment under cognitive workload is an obvious detriment to traffic safety. For example, Strayer et al. [270] found that recognition memory for objects in the driving environment was reduced by 50% when the driver was talking on a handsfree cell phone, inducing failures of visual attention during driving. Indeed, visual, manual and cognitive distraction often occur simultaneously while driving (e.g., texting while driving and other cell-phone reading and writing activities). Therefore, the estimates of crash risk based on comparisons of activities to just-driving baselines may need to be reconsidered in light of the possible finding that just-driving baselines may contain the aforementioned frequent cognitive activity. As a result, for example, secondary tasks effects while driving should be revised [269]. Accordingly, as detecting driver distraction depends on how distraction changes his/her behavior compared to normal driving without distraction, periods with minimal or no cognitive activity should be identified in order to train the distraction detection algorithms.

Additionally, computer vision techniques can be used, not only for extracting information inside the car, but also for extracting information outside the car, such as traffic, road hazards, external conditions of the road ahead, intersections, or even position regarding other cars. The final step should be the correlation between the driver’s understanding and the traffic context. One of the first works trying to fuse “out” information (visual lane analysis) and “in” information (driver monitoring) is the one proposed by Apostoloff et al. [271], pointing out the benefits of this approach. Indeed, visual lane analysis can be used for “higher-order tasks”, which are defined by interacting with other modules in a complex driver assistance system (e.g., understanding the driver’s attentiveness—distraction—to the lane-keeping task [272]). Hirayama et al [273] focused on temporal relationships between the driver’s eye gaze and the peripheral vehicles behaviour. In particular, they concluded that the timing when a driver gazes towards the overtaking event under cognitive distraction is later than that under the neutral state. Therefore, they showed that the temporal factor, that is, timing, of a reaction is important for understanding the state by focusing on cognitive distraction in a car-driving situation. Additionally, Rezaei et al. [87] proposed a system correlating the driver’s head pose to road hazards (vehicle detection and distance estimation) by analyzing both simultaneously. Ohn et al. [274] proposed a framework for early detection of driving maneuvers using cues from the driver, the environment and the vehicle. Tawari et al. [275] provided early detection of driver distraction by continuously monitoring driver and surround traffic situation. Martin et al. [276] focused on intersections and studied the interaction of head, eyes and hands as the driver approaches a stop-controlled intersection. In this line work of research, Jain et al. [277] deal with the problem of anticipating driving maneuvers a few seconds before the driver performs them.

There are many factors that can modulate distraction. For example, as discussed in Section 7, emotional information can modulate attention and decision-making abilities. Additionally, numerous studies link highly aroused stress states with impaired decision-making capabilities [278], decreased situational awareness [279], and degraded performance, which could impair driving ability [280]. Another driver state, often responsible for traffic violations and even road accidents that can lead to distraction, is confusion or irritation, as it is related to loss of self-control and, therefore, loss of vehicle control, which can be provoked by non-intuitive user interfaces or defective navigation systems as well as by complex traffic conditions, mistakable signs and complicated routing. Moreover, the amount of information that needs to be processed simultaneously during driving is a source of confusion especially for older people [281], who have slower perception and reaction times. Just like stress, confusion or irritation leads to impairment of driving capabilities including driver’s perception, attention, decision making, and strategic planning. Nervousness corresponds to a level of arousal above the “normal” one, which best suits to the driving task [211]. It is an affective state with negative impact both on decision-making process and strategic planning. Nervousness can be induced by a variety of reasons either directly related to the driving task like novice drivers or by other factors like personal/physical conditions [211].

The system should be validated, firstly, in a driver simulator and afterwards, in real conditions, where various factors including variations in lighting and noise can also affect both the driver’s attention and the performance of the developed algorithms. Therefore, public data sets covering simulated and real driving environments should be released. The driver’s physiological responses could be different in a driver simulator from those in real conditions [11,167,257,263]. Hence, while developing an inattention detection system, the simulated environment must be a perfect replica of the real environment. However, they are normally used in research and simulated scenarios, but not in real ones, due to the problems of vision systems working in outdoor environments (lighting changes, sudden movements, etc.). Moreover, they do not work properly with users wearing glasses and may need high computational requirements.

Data-driven applications will require large amount of labeled images for both training and testing the system. Both manual data reduction and labeling of data are time-consuming and they are also subject to interpretation of the reductionist. Therefore, to deal with this problem, two approaches are emerging from the literature: (1) unsupervised or semi-supervised learning and (2) automatic data reduction. For example, in connection with the first approach, Liu et al. [189] commented the benefits of SSL methods. Specifically, the explained the benefits of using SSL increased with the size of unlabeled data set showing that by exploring the data structure without actually labeling them, extra information to improve models performance can be obtained. On the other hand, there has been a hype in data reduction using vehicle dynamics and looking outside on large scale naturalistic driving data [282,283,284], and looking in at the driver [285].

In many distraction detection systems, the use of commercial sensors is usually performed [77,239,241,245,246,247]. We understand that the reason from this is twofold: these systems are well-established solutions offering both head and gaze tracking in the car environment and the efforts of the investigation can be focused to detect and predict distraction from the outputs from these commercial sensors instead of developing a new sensor from the very beginning. These commercial sensors can operate using one camera [239,245,246,247], two cameras [241] or even up to 8 cameras [77] placed all over the vehicle cabin. What we find missing is some research works trying to compare these commercial sensors in order to highlight the pros and cons of each one. Also, missing from the literature is the comparison between a new sensor and a commercial one trying to offer a competitive solution from the sake of the research community.

## Figures and Tables

**Table 1:** Mean Absolute Error (MAE) (in degrees) of face tracking algorithms comparison working in an automobile environment.

Algorithm	Roll(${}^{\circ }$)	Yaw(${}^{\circ }$)	Pitch(${}^{\circ }$)
La Cascia et al. [64]	9.8	4.7	2.4
Oyini et al. [56] average results 1 camera	5.3	3.9	5.2
Oyini et al. [56] uniform illumination 1 camera	4.8	3.8	3.9
Oyini et al. [56] varying illumination 1 camera	5.3	5.1	6.3
Vicente et al. [49] 1 camera	3.2	4.3	6.2
Pelaez et al. [60] 1 Kinect device	2.7	3.8	2.5
Murphy et al. [59] 1 camera	2.4	4.7	3.4
Tawari et al. [51] (MPS + POS) 1 camera	3.0	8.2	7.6
Tawari et al. [51] (MPS + POS) 2 cameras	3.8	7.0	8.6
Tawari et al. [51] (MPS + POS) 3 cameras	3.5	5.9	9.0
Tawari et al. [51] (CLM + POS) 1 camera	3.4	6.9	9.3
Tawari et al. [51] (CLM + POS) 2 cameras	3.6	5.7	8.8
Tawari et al. [51] (CLM + POS) 3 cameras	2.7	5.5	8.5

**Table 2:** Classification accuracy evaluated on the Southeast University (SEU) driving posture dataset [88].

Algorithm	Features	Classifier	Average Accuracy (%)
Zhao et al. [88]	Homomorphic filtering, skin-like regions segmentation and Contourlet Transform (CT)	RF	90.63
Zhao et al. [89]	Geronimo-Hardin-Massopust (GHM) multiwavelet transform	Multiwavelet Transform	89.23
Zhao et al. [90]	Histogram-based feature description by Pyramid Histogram of Oriented Gradients (PHOG) and spatial scale-based feature description	Perceptron classifiers	94.20
Zhao et al. [91]	Homomorphic filter, skin-like regions segmentation, canny edge detection, connected regions detection, small connected regions deletion and spatial scale ratio calculation	Bayes classifier	95.11
Bosch et al. approach [94]	PHOG	SVM	91.56
Lowe et al. approach [95]	SIFT	SVM	96.12
Yan et al. [93]		CNN	99.78

**Table 3:** Computer vision algorithms to detect cell phone usage. High recognition rates are usually obtained using very different approaches.

Algorithm	Features	Classifier	Recognition Rate (%)
Zhang et al. [96]	Features from the driver’s face, mouth and hand	Hidden Conditional Random Fields (HCRF)	91.20
Artan et al. [97]	Image descriptors extracted from a region of interest around the face	SVM	86.19
Berri et al. [98]	Percentage of the Hand and Moment of Inertia	FV	91.57
Xu et al. [99]	DPM	FV	95
Seshadri et al. [100]	Raw pixels and HOG features	Real AdaBoost, SVM, RF	93.86

**Table 4:** Hands recognition in different regions inside the car using CVRR-HANDS 3D dataset [106].

Algorithm	Features	Classifier	Regions	Recognition Rate (%)
Ohn et al. [106]	RGB data	SVM	5	52.1
Ohn et al. [106]	RGB combined with depth data	SVM	5	69.4
Martin et al. [104]	Hands cues	SVM	3	83
Martin et al. [104]	Hands and head cues	SVM	3	91
Ohn et al. [105]	Hands cues	SVM	3	90
Ohn et al. [105]	Hands and head cues	SVM	3	94

**Table 5:** AUC comparisons by algorithm across tasks.

Task	Algorithm			
	RVSP	EOFR	AttenD	MDD
Arrows	0.67	0.75	0.71	0.87
Bug	0.78	0.87	0.80	0.86

**Table 6:** Supervised algorithms for cognitive distraction detection.

Algorithm	Features	Classifier	Accuracy (%)
Zhang et al. [178]	Eye gaze-related features and driving performance	Decistion Tree	81
Zhang et al. [178]	Eye gaze-related features	Decistion Tree	80
Zhang et al. [178]	Pupil-diameter features	Decistion Tree	61
Zhang et al. [178]	Driving performance	Decistion Tree	60
Liang, Reyes, et al. [179]	Eye gaze-related features and driving performance	SVM	83.15
Liang, Reyes, et al. [179]	Eye gaze-related features	SVM	81.38
Liang, Reyes, et al. [179]	driving performance	SVM	54.37
Liang, Lee, et al. [180]	Eye gaze-related features and driving performance data	DBNs	80.1
Miyaji et al. [156]	Heart rate, Eye gaze-related features and pupil diameter	AdaBoost	91.5
Miyaji et al. [156]	Eye gaze-related features	SVM	77.1 (arithmetic task)
Miyaji et al. [156]	Eye gaze-related features	SVM	84.2 (conversation task)
Miyaji et al. [156]	Eye gaze-related features	AdaBoost	81.6 (arithmetic task)
Miyaji et al. [156]	Eye gaze-related features	AdaBoost	86.1 (conversation task)
Yang et al. [187]	Eye gaze-related features and driving performance data	ELM	87.0
Yang et al. [187]	Eye gaze-related features and driving performance data	SVM	82.9

**Table 7:** Mixing types of distraction detection algorithms.

Algorithm	Features	Classifier	Average Accuracy (%)
Li et al. [194]	AU and head pose	LDC (visual distraction) and SVM (cognitive distraction)	80.8 (LDC), 73.8 (SVM)
Craye et al. [195]	eye behaviour, arm position, head orientation and facial expressions using both color and depth images	Adaboot and HMM	89.84 (Adaboot), 89.64 (HMM)
Liu et al. [196]	Head and eye movements	SVM, ELM and CR-ELM	85.65 (SVM), 85.98 (ELM), 86.95 (CR-ELM)
Ragab et al. [197]	arm position, eye closure, eye gaze, facial expressions and head orientation using depth images	Adaboost, HMM, RF, SVM, CRF, NN	82.9 (RF—type of distraction detection), 90 (RF—distraction detection)

**Table 8:** Summary of visual-based approaches to detect different types of driver distraction.

Approach	Distraction Detection Approaches			Real Conditions	Operation	
	Manual	Visual	Cognitive		Daytime	Nighttime
Zhao et al. [88]	✔	✘	✘	✘	✔	✘
Zhao et al. [89]	✔	✘	✘	✘	✔	✘
Zhao et al. [90]	✔	✘	✘	✘	✔	✘
Zhao et al. [91]	✔	✘	✘	✘	✔	✘
Bosch et al. [94]	✔	✘	✘	✘	✔	✘
Lowe et al. [95]	✔	✘	✘	✘	✔	✘
Yan et al. [92]	✔	✘	✘	✘	✔	✘
Yan et al. [93]	✔	✘	✘	✘	✔	✘
Zhang et al. [96]	✔	✘	✘	✘	✔	✘
Artan et al. [97]	✔	✘	✘	✔	✔	✔
Berri et al. [98]	✔	✘	✘	✔	✔	✘
Xu et al. [99]	✔	✘	✘	✔	✔	✔
Seshadri et al. [100]	✔	✘	✘	✔	✔	✘
Ohn et al. [106]	✔	✘	✘	✔	✔	✘
Martin et al. [104]	✔	✘	✘	✔	✔	✘
Ohn et al. [105]	✔	✘	✘	✔	✔	✘
Morimoto et al. [120]	✘	✔	✘	✔	✘	✔
Ji et al. [121]	✘	✔	✘	✔	✘	✔
Ji et al. [122]	✘	✔	✘	✔	✘	✔
Ji et al. [123]	✘	✔	✘	✔	✘	✔
Gu et al. [124]	✘	✔	✘	✔	✘	✔
Batista el al. [125]	✘	✔	✘	✔	✘	✔
Bergasa et al. [126]	✘	✔	✘	✔	✔	✔
Lee et al. [114]	✘	✔	✘	✔	✔	✔
Vicente et al. [49]	✘	✔	✘	✔	✔	✔
Cyganek et al. [134]	✘	✔	✘	✔	✔	✔
Donmez et al. [142]	✘	✔	✘	✘	✔	✘
Klauer et al. [7]	✘	✔	✘	✔	✔	✔
Kircher et al. [143]	✘	✔	✘	✔	✔	✔
Kircher et al. [144]	✘	✔	✘	✔	✔	✔
Kircher et al. [145]	✘	✔	✘	✔	✔	✔
Victor et al. [146]	✘	✔	✘	✔	✔	✘
Zhang et al. [178]	✘	✘	✔	✘	✔	✘
Liang et al. [179]	✘	✘	✔	✘	✔	✘
Liang et al. [180]	✘	✘	✔	✘	✔	✘
Liang et al. [181]	✘	✘	✔	✘	✔	✘
Liang et al. [27]	✘	✘	✔	✘	✔	✘
Liang et al. [182]	✘	✘	✔	✘	✔	✘
Liang et al. [183]	✘	✘	✔	✘	✔	✘
Miyaji et al. [184]	✘	✘	✔	✘	✔	✘
Miyaji et al. [156]	✘	✘	✔	✘	✔	✘
Yang et al. [187]	✘	✘	✔	✘	✔	✘
Li et al. [194]	✘	✔	✔	✘	✔	✘
Craye et al. [195]	✔	✔	✘	✘	✔	✘
Liu et al. [196]	✘	✔	✔	✘	✔	✘
Ragab et al. [197]	✔	✔	✘	✘	✔	✘
